# Innovations in Robotic-Assisted Bronchoscopy: Current Trends and Future Prospects

**DOI:** 10.3390/diagnostics16060832

**Published:** 2026-03-11

**Authors:** Joshua M. Boster, S. Michael Goertzen, Brian D. Tran, Robert F. Browning

**Affiliations:** 1 Department of Medicine, Pulmonary Critical Care Medicine, Interventional Pulmonology, Walter Reed National Military Medical Center, Bethesda, MD 20889, USA; 2 Department of Pulmonology and Critical Care, Brooke Army Medical Center, Fort Sam Houston, San Antonio, TX 78234, USA; 3 Department of Pulmonology and Critical Care, University of California San Diego, San Diego, CA 92093, USA; briantran54@gmail.com

**Keywords:** robotic-assisted bronchoscopy, lung diseases, cancer, interventional pulmonology, cone-beam computed tomography, bronchoscopy, artificial intelligence

## Abstract

Robotic-assisted bronchoscopy (RAB) represents a significant technological advance, providing superior precision, enhanced visualization, and increased maneuverability relative to conventional bronchoscopic methods. This review provides an overview of current research evaluating RAB’s diagnostic performance and exploring future prospects. Recent literature demonstrates advantages in navigating difficult-to-reach lung lesions with improved safety profiles compared to transthoracic approaches. Incorporating advanced imaging technologies has enhanced real-time decision-making during procedures, and artificial intelligence applications are emerging. RAB has been rapidly adopted at many high-volume centers based on favorable navigational success and safety data. As the field matures, ongoing prospective studies will further define its role in improving patient outcomes, cost-effectiveness, and optimal integration with lung cancer screening programs. RAB faces ongoing challenges including substantial capital costs, training requirements, and need for standardized protocols. Therapeutic applications show promise and are under active investigation.

## 1. Introduction

The global burden of lung cancer remains substantial, with peripheral pulmonary lesions (PPLs) representing a growing diagnostic challenge in the era of widespread computed tomography (CT) based screening. Often these lesions are small and located within the outer third of the lung parenchyma making them challenging to reliably access using conventional bronchoscopic techniques. Conventional flexible bronchoscopy provides limited navigational reach beyond segmental bronchi and is associated with diagnostic yields as low as 30–50% for small peripheral lesions, particularly when the airway lacks a clear bronchus sign [1]. CT-guided transthoracic needle aspiration (TTNA), although associated with higher diagnostic yield, carries a significant complication profile, including pneumothorax rates approaching 15–25% in some cohorts [2]. The limitations of both conventional bronchoscopy and TTNA underscore the need for novel technologies that improve access to peripheral regions while maintaining a favorable safety profile. Robotic-assisted bronchoscopy (RAB) has emerged as a transformative technology to overcome the inherent limitations of conventional bronchoscopy. Leveraging enhanced catheter stability, precise catheter tip control, and integration with advanced imaging technologies, RAB platforms enable unprecedented reach into subsegmental airways with a recent study demonstrating a 98.7% success rate for reaching target PPLs [3]. As lung cancer diagnosis and management evolves toward minimally invasive and highly targeted approaches, RAB is poised to play a central role in diagnosis, staging, and potentially localized therapy. This review provides an overview of current evidence on RAB, focusing on technological advances, diagnostic performance, clinical outcomes, and emerging applications.

## 2. Platform Evolution and Comparative Performance: Technical and Methodological Considerations

Early efforts to improve access to PPLs relied on techniques such as fluoroscopic guidance, virtual bronchoscopy, and electromagnetic navigation bronchoscopy (ENB) [4]. Despite these advances, these modalities still faced major limitations, including poor catheter stability, CT-to-body divergence (CTBD), suboptimal diagnostic performance, and the lack of tool-in-lesion visualization. The introduction of robotic platforms represented a significant technological advance to mitigate those limitations. Currently three United States Food and Drug Administration (FDA) 510(k) cleared RAB platforms are available including the Monarch Robotic Bronchoscopy System (Johnson & Johnson) FDA cleared March 2018, Ion Robotic Bronchoscopy System (Intuitive Surgical) FDA cleared February 2019, and The Galaxy System (Noah Medical) FDA cleared March 2023 [5]. The three FDA-cleared RAB platforms vary substantially in technical specifications, navigation technology, and imaging integration capabilities (Table 1). These differences may influence both diagnostic performance and institutional platform selection. The Monarch^TM^ and Galaxy^TM^ platforms utilize electromagnetic navigation technology, while the Ion^TM^ platform employs fiber optic shape-sensing technology. All platforms are compatible with adjunctive imaging such as radial endobronchial ultrasound (rEBUS) and cone-beam CT (CBCT), although they differ in navigational approach and imaging integration. Although theoretical advantages exist for each platform, comparative data are extremely limited.

Currently, the only available head-to-head comparison of RAB platforms is a single-center retrospective study by McNierney et al., which reported higher diagnostic yield with Ion (84.2%) compared to Monarch (71.0%, *p* = 0.003), alongside shorter procedure times and reduced radiation [6]. However, significant methodological challenges limit definitive conclusions regarding relative platform performance. Specifically, the diagnostic outcomes in this study reflect a shifting “procedural ecosystem” rather than platform technology alone; 95% of Ion procedures (193/203) utilized a 1.1 mm (mm) cryoprobe, whereas only 6% of Monarch procedures (10/162) employed this technique. This disparity reflects the rapid institutional adoption of cryobiopsy techniques between 2021 and 2023, following the development of 1.1 mm probes that allow through the channel retrieval which avoids the safety risks and navigational loss associated with traditional en bloc scope removal [7,8,9,10,11]. Given that cryobiopsy has been reported to improve diagnostic yield by 15–25% over forceps alone the superior outcomes reported by McNierney et al. may reflect this shift in sampling technique [7,10,12,13]. Furthermore, the sequential implementation of the platforms (Monarch 2021–2023; Ion 2023) introduced additional confounders, including Ion’s integrated CBCT with real-time target updating versus Monarch’s non-integrated CBCT, as well as evolving operator experience. Consequently, these results likely reflect the maturation of institutional RAB practice and sampling technology rather than a primary difference in platform design, highlighting the need for prospective trials with standardized sampling protocols.

## 3. Diagnostic Performance and Comparative Effectiveness of RAB

Conventional flexible bronchoscopy has historically shown limited diagnostic performance for PPLs less than 2 cm (cm). These limitations drove the development of navigational platforms, like ENB, which improved access to peripheral targets but continued to show variable diagnostic yields across studies ranging from 65 to 77% with performance strongly influenced by lesion size, the presence of a bronchus sign, and the use of adjunctive imaging modalities [14,15]. A primary drawback of ENB alone, in comparison to RAB, is the lack of catheter stability and catheter articulation that requires manual manipulation which limits reach into smaller peripheral airways and makes precise positioning of biopsy instruments challenging. In contrast, RAB platforms address many of these shortcomings, enabling greater stability, more precise movements, and improved access to distal airways. Despite the advantages associated with RAB, there are limited prospective comparative studies directly comparing ENB to RAB, and the interpretation of retrospective studies is complicated by substantial heterogeneity in study design, variable definitions of diagnostic yield, and variable use of adjunctive imaging. Many early ENB studies predated routine use of CBCT and other advanced imaging modalities, whereas most contemporary RAB series incorporate these tools, potentially inflating apparent yield differences. At the time of this review, the RELIANT trial, is the only available prospective randomized comparison and demonstrated that the diagnostic yield of RAB was non-inferior to that of ENB (77.8% vs. 75.5%) [16]. In that study, the ENB cohort included integrated digital tomosynthesis for intraprocedural imaging correction, while the robotic cohort did not have integrated CBCT which likely influenced the results. This potential influence is highlighted by a retrospective study which demonstrated that when CBCT was utilized for both RAB and ENB, the diagnostic yield of RAB-CBCT was significantly higher (89.2% vs. 66% *p* < 0.001) [17]. Key prospective and comparative trials evaluating RAB diagnostic performance are summarized in (Table 2). Beyond the McNierney study, comparative evidence for RAB platforms remains limited, with most published data consisting of single-arm observational studies of individual platforms. The TARGET trial evaluated RAB (primarily Monarch) in a multicenter prospective registry, reporting 73.1% diagnostic yield for pulmonary nodules [3]. Similarly, a prospective multicenter study of the Ion platform by Kalchiem-Dekel et al. had an 81.7% diagnostic yield in 131 patients [18]. However, direct comparison of these results is complicated by several interrelated factors that introduce systematic bias and limit valid cross-study comparisons. A comparison of diagnostic performance and safety profiles across conventional bronchoscopy, ENB, RAB, and TTNA is provided in (Table 3). Operator experience and institutional learning curves may play a critical role in RAB outcomes. Procedural competency in related bronchoscopic procedures exhibits both an initial learning curve and volume-dependent outcome improvement [19]. The sequential implementation of platforms in comparative studies may introduce systematic bias, as operators gain experience with navigational techniques, patient selection, and procedural workflows that benefit subsequent platform evaluations regardless of technological differences. Additionally, the integration of complementary technologies varies substantially across studies and institutions. Advanced imaging modalities, particularly CBCT, have shown substantial improvements in diagnostic yield when integrated with navigational bronchoscopy, with some studies reporting 15–40% relative increases in diagnostic success [20,21,22,23,24,25]. Studies have shown that CTBD occurs in up to 67% of cases during electromagnetic navigation, necessitating real-time imaging correction for optimal targeting [26,27]. The extent and sophistication of CBCT integration varies across RAB platforms and continues to evolve, making it challenging to isolate platform-specific performance from imaging-related benefits. Integrated CBCT systems with automated target updating, such as those employed in the Ion platform, enable dynamic correction of CTBD during the procedure. The recently FDA-cleared Monarch QUEST system (2025) now facilitates CBCT integration for the Monarch platform [28]. In contrast, non-integrated systems require manual operator interpretation and adjustment. Similarly, the choice of sampling instrumentation including the adoption of ultrathin cryoprobes as discussed previously represents a major determinant of diagnostic outcomes that may vary independently of platform selection. The synergistic effects of platform technology, imaging integration, and sampling tools create a complex system where individual component contributions are difficult to disaggregate. Finally, long-term outcome data beyond diagnostic yield including impact on time-to-treatment, avoidance of invasive procedures, and cost-effectiveness remain sparse for all RAB platforms. While the technology offers theoretical advantages in enabling earlier diagnosis and reducing the need for surgical biopsy, prospective comparative studies with meaningful clinical endpoints are needed to validate these assumptions. The rapid evolution of both platform technology and complementary techniques suggests that contemporary practice may differ substantially from earlier published series, necessitating ongoing reassessment of comparative effectiveness as the field matures.

Historically, TTNA has been considered the gold standard for the diagnosis of PPLs particularly for smaller lesions, with an excellent diagnostic yield but suboptimal rate of complications including relatively high rates of pneumothorax and pulmonary hemorrhage [29]. The risk for complications with TTNA is even higher in patients with emphysema, deeper lesions, smaller nodules, and when multiple passes are required [30,31,32]. There is a paucity of prospective data directly comparing ENB and RAB to TTNA. At the time of this review, the VERITAS trial is the only prospective randomized controlled trial comparing ENB to TTNA for the diagnosis of small PPLs. In that study, the diagnostic yield of ENB was non-inferior to TTNA (79% vs. 73.6% *p* = 0.003) and the rate of complications was significantly less in the ENB cohort (5% vs. 29.2% *p* < 0.001), particularly in regard to pneumothorax (3.3% vs. 28.3% *p* < 0.001) [33]. There was no significant difference in the rate of other complications including hemorrhage. While the VERITAS trial used ENB rather than a robotic platform, these findings highlight the evolving role of advanced bronchoscopic techniques for the diagnosis of small PPLs. However, several methodological considerations merit attention when interpreting these results. The VERITAS trial has been critiqued for the underutilization of rapid on-site evaluation (ROSE) in the TTNA arm, which likely disadvantaged its performance and may have contributed to the observed yield differences [34]. Additionally, important technical differences exist between ENB and RAB that may influence comparative outcomes. RAB procedures are uniformly performed under general anesthesia with neuromuscular blockade and endotracheal intubation, enabling complete patient immobilization and optimized breath-hold maneuvers during intraprocedural imaging [35,36,37,38]. This motionless state reduces CTBD and motion artifact while specialized ventilation protocols with differential PEEP minimize atelectasis formation, particularly in dependent lung regions [35,36]. Recent evidence demonstrates that lateral decubitus positioning may be superior to ventilatory strategies alone in preventing atelectasis from obscuring targets during robotic bronchoscopy, further improving procedural outcomes [39]. In contrast, ENB in the VERITAS trial utilized moderate sedation in most cases, and TTNA continues to be performed predominantly under local anesthesia with conscious sedation rather than general anesthesia [34]. The combination of robotic catheter stability, general anesthesia with paralysis, and breath-hold capabilities in RAB may provide advantages over both ENB and conventional TTNA approaches, though direct comparative trials are needed to confirm this hypothesis. Currently no prospective randomized trials have directly compared RAB to TTNA. Despite the assumed non inferiority or superiority of RAB to conventional bronchoscopy with ENB, future studies evaluating RAB-CBCT combinations with optimized anesthesia protocols in comparison to TTNA are needed to demonstrate diagnostic yields comparable to or exceeding those of TTNA while maintaining the markedly lower complication rates demonstrated in the VERITAS trial with the use of ENB. In light of these findings, the traditional paradigm which often prioritized TTNA for diagnostic yield in peripheral nodules may need reconsideration.

## 4. Artificial Intelligence and Automation in RAB

Currently, FDA cleared RAB platforms rely primarily on traditional navigation technologies including electromagnetic tracking and fiber-optic shape sensing, without integration of artificial intelligence for core navigation functions. However, emerging AI applications demonstrate potential to substantially advance RAB capabilities by enhancing procedural planning, improving navigation precision, and augmenting operator performance. As robotic systems generate increasingly detailed procedural data including catheter trajectories, airway visualization sequences, and tool-lesion interactions AI algorithms can leverage these large datasets to identify patterns associated with successful outcomes and provide real-time guidance tailored to individual patient anatomy and lesion characteristics. Currently FDA-cleared RAB platforms incorporate limited algorithmic assistance for pathway suggestion but do not yet integrate adaptive machine learning capabilities. Although AI-enhanced RAB demonstrates substantial theoretical promise in multiple domains, the supporting clinical evidence remains limited, with most studies conducted in controlled experimental settings. Among potential applications, AI-assisted navigation and operator augmentation represent particularly active areas of investigation. Zhang and colleagues developed an AI co-pilot bronchoscope robot utilizing an AI-human shared control algorithm trained on historical bronchoscopic videos and expert demonstrations [40]. The system features a plug-and-play robotic catheter (available in 3.3 mm and 2.1 mm diameters) capable of accessing bronchi beyond the fifth generation in average adult patients. This AI-human collaborative approach enables the AI system to predict optimal steering actions (pitch and yaw angles) based on bronchoscopic images combined with coarse-grained human commands (directional inputs such as up, down, left, right, or forward), while actively preventing misoperation that could damage airway walls. The system was validated through both in vitro phantom studies and in vivo testing in a minipig model, where novice operators using AI assistance achieved navigation performance and safety profiles comparable to experienced bronchoscopists. By maintaining the bronchoscope in the center of the airway in real-time, the AI co-pilot reduced collisions with airway walls and ensured an unobstructed field of view throughout navigation. Complementing this shared-control approach, Banach and colleagues investigated conditional autonomy in robot-assisted bronchoscopy through a system that automates catheter advancement while preserving high-level human decision-making [41]. In this paradigm, operators specify the next target airway at bronchial bifurcation points, and the autonomous system then navigates and aligns the bronchoscope to that location using only monoscopic bronchoscopic video as input. The system was validated through in vitro, ex vivo, and in vivo testing, demonstrating that conditional automation reduced average navigation time between airway segments compared to manual human operation (1.3 s vs. 2.5 s per segment). While this improvement in navigation speed represents a modest efficiency gain, the primary value lies in the system’s ability to execute consistent, smooth catheter movements that minimize tissue trauma while freeing operators to focus on higher-level procedural decisions such as pathway selection and biopsy planning. These early investigations highlight the potential for AI-assisted and semi-autonomous navigation to enhance procedural efficiency, reduce operator-dependent variability, and improve safety through more consistent catheter control. By lowering the technical threshold required for competent performance, AI augmentation could shift RAB from being a highly operator-dependent technology to a more standardized and reproducible intervention. This democratization of expertise may accelerate dissemination of RAB into community hospitals and lower-volume centers that lack experienced interventional pulmonologists, potentially addressing healthcare disparities in access to advanced bronchoscopic diagnosis. However, several critical questions remain unanswered. First, the extent to which improved navigation performance translates into meaningful improvements in diagnostic yield, complication rates, and patient-centered outcomes requires validation in prospective clinical trials. Second, the optimal balance between human control and autonomous assistance ranging from purely advisory systems to conditional autonomy to full automation remains unclear and may vary based on procedural complexity, operator experience, and institutional preferences. Finally, regulatory frameworks for AI-integrated medical devices, requirements for algorithm validation and generalizability across diverse patient populations, and strategies for continuous learning and algorithm updating in clinical practice require further development before widespread clinical implementation.

## 5. Clinical Impact and Health Economics

Historically, lung cancer has been diagnosed at advanced stages in the majority of cases, with only a small fraction identified at an early stage, when the disease is more treatable and potentially curable. In 2016, only 16% of lung cancers were diagnosed at a localized stage in the United States [42]. This trend has been improving over time but remains suboptimal. Emerging evidence suggests that RAB may be associated with improved diagnosis of early-stage lung cancer and has the potential to continue shifting diagnosis earlier, thereby potentially enabling timely curative intent therapy and improving downstream oncologic outcomes. In a recently published propensity-matched retrospective cohort study comparing lung cancer diagnoses made by RAB vs. TTNA, the RAB cohort was more frequently diagnosed at an early stage compared to the TTNA group (OR = 3.02, 95% CI: 1.83–5.04, *p* < 0.001) [43]. This single-center study compared sequential rather than concurrent cohorts (TTNA: 2017–2020; RAB: 2020–2023), introducing similar temporal confounding concerns as previously discussed regarding the evolution of practice patterns and external factors such as COVID-19 and expanded lung cancer screening guidelines. This occurred despite comparable diagnostic yields for malignancy between the two approaches. Several factors may explain this observed stage shift beyond the diagnostic modality itself. First, the improved safety profile of RAB may encourage biopsy of smaller or less accessible lesions that might otherwise have been managed with surveillance, potentially introducing length-time bias favoring detection of slower-growing tumors. Second, detection of cancer at an earlier stage (lead-time bias) does not necessarily translate to improved survival if the natural disease course remains unchanged. Third, RAB’s ability to perform concurrent nodal staging during the same procedure may provide more complete baseline staging information compared to TTNA patients who underwent sequential staging, confounding the stage-at-diagnosis comparison. Despite this theoretical advantage, there is currently no published data comparing the time-to-treatment between RAB and TTNA for lung cancer patients.

As RAB adoption is relatively recent, long-term survival data are still maturing and will be essential to fully characterize the clinical impact of earlier-stage detection on patient outcomes.

Although RAB platforms require substantial capital investment, emerging preliminary cost-effectiveness analyses suggest potential financial benefit in specific healthcare settings when accounting for downstream healthcare utilization [5]. However, rigorous health economic analyses accounting for all relevant costs and outcomes remains limited. The safety profile of RAB results in significantly lower hospitalization rates compared to TTNA (5.41% vs. 19.59%, *p* < 0.001) in the previously discussed single-center study [43]. While reduced hospitalization may suggest cost savings in certain healthcare systems, this metric alone does not constitute a formal cost-effectiveness analysis. Comprehensive economic evaluation would require accounting for direct procedural costs (equipment, OR time, anesthesia, consumables), capital amortization and maintenance costs, downstream costs (treatment, surveillance, complications), and opportunity costs. Catheter costs vary by platform: some systems utilize reusable catheters with limited reprocessing cycles, while others employ single-use disposables, each with distinct economic implications. Furthermore, incorporating endobronchial ultrasound (EBUS) staging into the same procedural session as RAB may increase both the clinical and economic value of RAB compared with TTNA. In contrast, a TTNA-based diagnostic strategy typically necessitates a separate staging procedure, adding delays, costs, care coordination complexity, patient inconvenience, and additional anesthetic risk. However, this comparison must be balanced against several considerations: (1) RAB uniformly requires general anesthesia while TTNA can often be performed with conscious sedation, (2) not all patients require mediastinal staging, and (3) the incremental benefit of same-session staging must be weighed against longer anesthesia time and its associated risks. Limited studies have suggested that the combination of RAB with surgical resection during a single anesthetic event is feasible and may reduce costs [44,45,46]. In one single-center study, the combined RAB and surgical resection cohort had reduced total operating time (459 min for combined vs. 513 min for standard) along with reported lower direct and indirect costs; however, actual cost data were not provided, and generalizability to other institutions remains uncertain [47].

In summary, emerging data suggest RAB may offer advantages in specific healthcare delivery models, particularly those with high-volume programs and established multidisciplinary infrastructure. While the evidence base continues to develop, preliminary findings regarding safety profiles and potential for integrated staging are encouraging.

## 6. Future Directions

Future advances in RAB will likely focus on improving reach, precision, and procedural efficiency while expanding the clinical scope of robotic platforms. Theoretical improvements in catheter flexibility and miniaturization may enable more reliable access to subsegmental and sub-subsegmental airways, though engineering challenges related to maintaining image quality, tool compatibility, and structural integrity in ultra-thin catheters remain significant. Smaller diameter robotic catheters, improved articulation mechanisms, and enhanced steering algorithms represent active areas of device development, with the potential to reduce airway trauma while improving positional stability at the target lesion. The incorporation of haptic feedback technologies has been proposed to augment operator awareness of tissue interaction, though evidence demonstrating clinical benefit in bronchoscopy is currently lacking. AI represents an emerging area of interest in RAB, with early-stage research exploring applications including prediction of optimal airway paths, automated catheter stabilization, adaptive navigation in response to respiratory motion, and alerts to potential safety risks. However, these AI-enabled capabilities remain largely conceptual, and robust clinical validation studies are needed before widespread implementation.

Beyond peripheral nodule biopsy, there is growing interest in the enhanced stability and precision of robotic platforms as a potential platform for therapeutic intervention. Very early feasibility studies are exploring the use of robotic platforms for the precise delivery of local therapies, including microwave and radiofrequency ablation, cryoablation, and targeted drug/nanoparticle delivery [48,49,50,51,52]. However, these applications remain investigational, with limited published data on safety, efficacy, and long-term oncologic outcomes. By leveraging the robotic catheter’s stability and integrating advanced imaging, it is theorized that bronchoscopists may be able to achieve precise TIL placement to ensure adequate and effective treatment margins during ablative procedures. The concept of RAB potentially serving as a unified platform for the diagnosis, staging, and definitive management of select early-stage lesions is intellectually compelling, as it could theoretically facilitate both diagnosis and treatment within a single anesthetic session. If proven safe and effective, this approach might reduce procedural burden, delays in care, and overall healthcare costs by eliminating the wait time typically found between biopsy and surgical resection or radiation. However, this vision remains aspirational, and critical questions regarding patient selection criteria, long-term oncologic outcomes, safety profiles, reimbursement models, and regulatory pathways must be addressed through rigorous clinical investigation before bronchoscopic ablation can be considered standard of care.

As RAB matures as a clinical technology, prospective multicenter studies and randomized controlled trials will be essential to rigorously evaluate diagnostic performance, complication rates, cost-effectiveness, and optimal workflow integration across diverse practice settings. Important areas for continued investigation include long-term clinical outcomes, health economic evaluations, standardized training and quality benchmarks, and real-world safety data across diverse practice settings. In addition, real-world data from registries and integration with lung cancer screening programs will be critical to understanding how RAB influences diagnosis patterns, time to treatment, and long-term clinical outcomes. Addressing these questions will help optimize RAB’s integration into clinical practice and fully define its evolving role in lung cancer diagnosis and management.

## 7. Conclusions

RAB represents a major advancement in the diagnosis and management of peripheral pulmonary lesions. Platform stability and precise control enable enhanced navigational success and diagnostic yield compared with conventional bronchoscopy, while multimodal imaging integration optimizes lesion targeting. RAB maintains a favorable safety profile relative to transthoracic needle aspiration, with the added advantage of concurrent mediastinal staging unavailable through percutaneous approaches. Many high-volume centers have adopted RAB as the preferred modality for PPL biopsy, recognizing these favorable performance characteristics. A growing body of clinical evidence, while consisting primarily of single-center studies and early-phase trials, supports RAB’s clinical utility across multiple platforms. Navigational success rates and safety profiles consistently favor RAB over traditional approaches. Ongoing challenges include capital costs that may limit access to community hospitals and lower-resource settings, need for standardized training and competency assessment, and continued workflow optimization. Despite these considerations, the field is rapidly evolving. Ongoing technological innovation, increasing institutional experience, and a growing body of clinical evidence support the continued evolution of RAB toward a more central role in lung cancer diagnosis. As future platforms expand into therapeutic applications, RAB has the potential to reshape the diagnostic-therapeutic continuum of lung cancer care.

Disclaimer: The views expressed in this article are those of the authors and do not necessarily reflect the official policy of the Department of Defense or the U.S. Government. The identification of specific products or scientific instrumentation is considered an integral part of the scientific endeavor and does not constitute endorsement or implied endorsement on the part of the authors, DoD, or any component agency.

## Figures and Tables

**Table 1:** Comparison of FDA-Cleared Robotic-Assisted Bronchoscopy Platforms.

Feature	Monarch (Auris/J&J)	Ion (Intuitive)	Galaxy (Noah Medical)
FDA Clearance	March 2018	February 2019	March 2023
Navigation Technology	Electromagnetic	Fiber-optic shape-sensing	Electromagnetic
Outer Diameter	4.4 mm	3.5 mm	~4.2 mm
Working Channel	2.0 mm	2.0 mm	2.0 mm
Scope Design	Scope-in-sheath	Single reusable robotic catheter (5 uses)	Single-use disposable
Integrated CBCT	Yes (Quest system with GE OEC 3D)	Yes (Siemens Cios Spin)	No (uses TiLT + technology)
Continuous Vision	Yes	No (requires probe removal)	Yes (always-on camera)
Irrigation/Aspiration	Yes	No	Yes
Tool-in-Lesion Verification	CBCT, digital tomosynthesis	CBCT	TiLT + tomosynthesis
Articulation	4-way, 180°	180° all directions	4-way, 180°

**Table 2:** Key Clinical Trials in Robotic-Assisted Bronchoscopy Development.

Trial	Year	Design	Platform	N	Primary Outcome	Key Finding
**TARGET**	2025	Prospective multicenter	Ion	300+	Safety and diagnostic yield	98.7% lesion reach rate; 89% diagnostic yield
**RELIANT**	2025	Prospective RCT	RAB vs. ENB	479	Diagnostic yield	RAB non-inferior to ENB (77.8% vs. 75.5%)
**VERITAS**	2025	Prospective RCT	ENB vs. TTNA	400+	Diagnostic accuracy	ENB non-inferior to TTNA (79% vs. 73.6%); fewer complications (5% vs. 29.2%)
**CONFIRM**	2025	Prospective multicenter	Ion + CBCT	150+	Tool-in-lesion rate	99.4% TIL confirmation; 89% diagnostic yield for small nodules
**FRONTIER**	2024	First-in-human	Galaxy + TiLT+	19	Feasibility and safety	100% navigation success; 89.5% diagnostic yield; 100% TIL accuracy
**McNierney**	2025	Retrospective propensity-matched	Ion vs. Monarch	304	Diagnostic yield	Ion superior to Monarch (84.2% vs. 71%; *p* = 0.003); shorter procedure time
**MATCH 2**	2026	Prospective observational	Galaxy + TiLT confirmed with CBCT	31	Tool in lesion rate, TiLT vs. CBCT correlation, Diagnostic yield	TIL 100% TiLT 97% Diagnostic yield 97%

Abbreviations: CBCT, cone-beam computed tomography; ENB, electromagnetic navigation bronchoscopy; N, sample size; RAB, robotic-assisted bronchoscopy; RCT, randomized controlled trial; TIL, tool-in-lesion; TiLT, Tool-in-Lesion Tomosynthesis; TTNA, transthoracic needle aspiration.

**Table 3:** Diagnostic Performance Comparison Across Biopsy Modalities.

Modality	Diagnostic Yield	Pneumothorax Rate	Study/References
Conventional Bronchoscopy (PPL < 2 cm)	30–50%	<2%	Meta-analysis [1]
ENB alone	65–77%	3–5%	Meta-analysis [7,8]
ENB (RELIANT trial)	75.5%	Not reported	RELIANT [9]
ENB (VERITAS trial)	79%	3.3%	VERITAS [15]
ENB with CBCT	66%	Not reported	Abdelghani et al. [10]
RAB without CBCT	50–77%	2–4%	Various studies [18,19,20]
RAB (RELIANT trial)	77.8%	Not reported	RELIANT [9]
RAB—Monarch	71%	2.6%	McNierney et al. [6]
RAB—Ion	84.2%	3.3%	McNierney et al. [6]
RAB with CBCT—Ion + Cios Spin	89%	<5%	CONFIRM [21]
RAB with CBCT—Ion	89.2%	Not reported	Abdelghani et al. [10]
RAB—Galaxy with TiLT+	89.5%	Not reported	FRONTIER [20]
RAB—Galaxy with TiLT confirmed with CBCT	97%	3%	MATCH 2 [22]
TTNA (VERITAS trial)	73.6%	28.3%	VERITAS [15]
TTNA (historical)	85–95%	15–25%	Meta-analysis [2,11,12,13,14]

## Data Availability

No new data were created or analyzed in this study. Data sharing is not applicable to this article.
